# Tuning Neuromodulation Effects by Orientation Selective Deep Brain Stimulation in the Rat Medial Frontal Cortex

**DOI:** 10.3389/fnins.2018.00899

**Published:** 2018-12-13

**Authors:** Lauri J. Lehto, Pavel Filip, Hanne Laakso, Alejandra Sierra, Julia P. Slopsema, Matthew D. Johnson, Lynn E. Eberly, Walter C. Low, Olli Gröhn, Heikki Tanila, Silvia Mangia, Shalom Michaeli

**Affiliations:** ^1^Center for Magnetic Resonance Research, University of Minnesota, Minneapolis, MN, United States; ^2^First Department of Neurology, Faculty of Medicine, St. Anne’s Teaching Hospital, Masaryk University, Brno, Czechia; ^3^A.I. Virtanen Institute for Molecular Sciences, University of Eastern Finland, Kuopio, Finland; ^4^Department of Biomedical Engineering, University of Minnesota, Minneapolis, MN, United States; ^5^Division of Biostatistics, University of Minnesota, Minneapolis, MN, United States; ^6^Department of Neurosurgery, University of Minnesota, Minneapolis, MN, United States

**Keywords:** deep brain stimulation, infralimbic cortex, fMRI, orientation selective, depression

## Abstract

Previous studies that focused on treating major depressive disorder with conventional deep brain stimulation (DBS) paradigms produced inconsistent results. In this proof-of-concept preclinical study in rats (*n* = 8), we used novel paradigms of orientation selective DBS for stimulating the complex circuitry crossing the infralimbic cortex, an area considered analogous to human subgenual cingulate cortex. Using functional MRI at 9.4 T, we monitored whole brain responses to varying the electrical field orientation of DBS within the infralimbic cortex. Substantial alterations of functional MRI responses in the amygdala, a major node connected to the infralimbic cortex implicated in the pathophysiology of depression, were observed. As expected, the activation cluster near the electrode was insensitive to the changes of the stimulation orientation. Hence, our findings substantiate the ability of orientation selective stimulation (OSS) to recruit neuronal pathways of distinct orientations relative to the position of the electrode, even in complex circuits such as those involved in major depressive disorder. We conclude that OSS is a promising approach for stimulating brain areas that inherently require individualisation of the treatment approach.

## Introduction

Despite best efforts of psychiatrists, the results of the mainstay treatment strategies in major depressive disorders (MDD) are often disappointing, labeling nearly a third of patients as therapy-refractory (Warden et al., 2007). This stalemate situation is not surprising, considering our lack of deeper insight in the exact neurophysiology of MDD. However, over the years several neural nodes have emerged as possible major culprits (Koenigs and Grafman, 2009), paving a path for possible targeted, even curative interventions, with high hopes invested mainly in deep brain stimulation (DBS) therapy. Since its renaissance in the 1990s, DBS has confidently risen to the position of a safe and effective therapeutic option for various movement disorders, epilepsy and even obsessive-compulsive disorder (Greenberg et al., 2006; Bari et al., 2018). Yet, the encouraging results yielded in several small, open-label MDD DBS studies targeting various structures failed to be replicated in two randomized, sham-controlled trials (Dougherty et al., 2015; Youngerman and Sheth, 2017). However, this was recently countered by another randomized clinical trial proving MDD DBS efficacy in the anterior limb of the internal capsule (Bergfeld et al., 2016). These disparate results call for better understanding not only of the targeted neural circuitry, but also of the interindividual variability in clinical and cognitive phenotypes (Widge et al., 2016), which may be directly relevant for optimizing the stimulation parameters and the implantation site (Morishita et al., 2014).

Advances in electrode design (Tsai et al., 2015) and stimulation paradigms (Martens et al., 2011; Chaturvedi et al., 2012) are promising venues for expanding and optimizing the stimulation outcomes. In particular, the recently introduced paradigms of orientation selective stimulation (OSS) (Lehto et al., 2017b) generate orientation dependent electric field gradients based on multichannel leads with independently driven channels, and can provide optimal stimulation of axonal pathways with distinct orientations relative to the position of the electrode. OSS was shown to be effective in more selectively stimulating axonal pathways crossing a highly organized structure such as the corpus callosum, but its effectiveness in more complex, cognitive-related circuitry has not been investigated.

Here, we aimed at characterizing, by fMRI, the involvement of neural circuitry during real-time DBS of a rat brain region that is relevant to major depression. Despite the considerable variations of the anatomy of the prefrontal cortex across species, and hence the somewhat controversial correspondence of various structures (Heidbreder and Groenewegen, 2003), the cytoarchitectonics and anatomical connections of the ventral aspect of the medial prefrontal cortex in rats are homologous to the sgACC in humans. More specifically, the infralimbic subregion (IL) has been implied in the mechanisms of stress (Diorio et al., 1993; Ostrander et al., 2003), and is one of the main candidates in MDD DBS in humans (Takagishi and Chiba, 1991; Gabbott et al., 2003; Hamani et al., 2010b). In addition, sgACC/IL is a good example of crossroads of several major fiber tracts. Within one hemisphere strong connections are sent to the mFC, medial thalamus, NAc, amygdala and hypothalamus. In addition, the ascending serotonergic and noradrenergic pathways pass through the sgACC/IL before diverging all over the cortical mantle. Undoubtedly, conventional electric stimulation even with bipolar electrodes would touch all these connections and thus influence all these networks to a variable extent (Hamani et al., 2011). The objective of this study was to compare the network-level responses to infralimbic DBS across multiple electric field orientations employing OSS and across multiple DBS frequencies. We hypothesized that OSS would titrate the fMRI responses in the ipsilateral limbic system, including the amygdala and NAc.

## Materials and Methods

### Animals

For DBS-fMRI, Sprague-Dawley rats (Envigo, Madison, WI, United States; *n* = 8, male, 295–327 g) were housed in pairs in a temperature and humidity-controlled vivarium with a 12-h light-dark cycle with food and water *ad libitum*. These animal procedures were approved by the Institutional Animal Care and Use Committee (IACUC) of the University of Minnesota.

### Electrode Implantation

**FIGURE 1:**
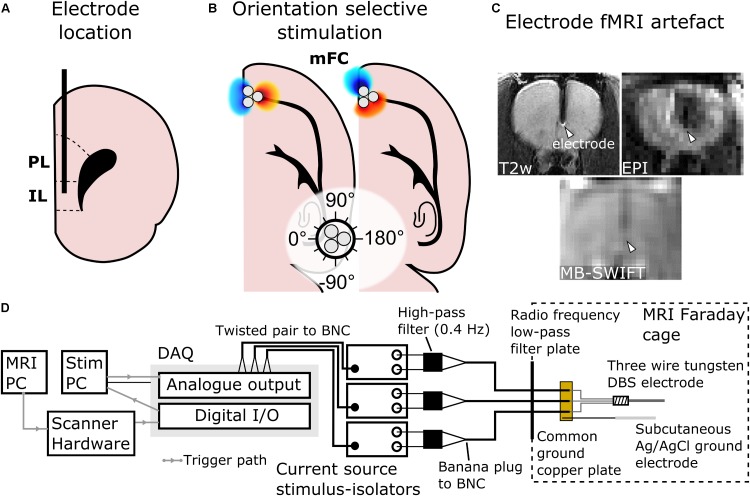
Schematic representation of the main features of the study design, including site of electrode implantation in IL. **(A)** Placement of the electrode in IL (PL, prelimbic cortex) shown in a coronal schematic drawing. **(B)** 0° and 60° orientations of dipoles in OSS to target different fibers, depicted in a horizontal schematic drawing. In this study, the stimulation angles 0° and 180° were chosen to be in the mediolateral direction and angles 90° and –90° were chosen to be in the rostrocaudal direction (white circle). **(C)** Metallic electrodes interfere with the magnetic field used for MRI leading to a susceptibility artifact (white arrowheads). The artifact is especially severe when using traditional Echo Planar Imaging (EPI) fMRI, whereas MB-SWIFT technique provides artifact-free fMRI. **(D)** Diagram of the stimulation system.

After the induction of isoflurane anesthesia (5% induction, 2–3% maintenance; carrier gas O_2_/N_2_O 30/70%), the animals were placed on a heating pad and into a stereotactic frame (Stoelting, Wood Dale, IL, United States). Temperature was monitored via a rectal probe and maintained at 37°C, while respiration rate was monitored using a plastic pressure sensor and maintained at 70–80 per min. Burr hole with a diameter of 0.7 mm was performed over the implantation target and a tripolar electrode composed of tightly braided three polyimide-insulated (30 μm thickness) tungsten electrodes (PlasticsOne, MS333T/3C-C 3TW, Roanoke, VA, United States) with tip-only contact diameter of 127 μm was inserted unilaterally in the right IL (Figure 1A; rostrocaudal 3.0 mm, mediolateral 0.6 mm and dorsoventral 5.2 mm). The orientation of the electrode tip (Figure 1B) was controlled using a microscope, identifying each channel with a multi-meter. The burr hole around the electrode was filled with gelatin foam (SPONGOSTAN^TM^, Søborg, Denmark) and covered using dental acrylic (Lang Dental, Jet Acrylic, Wheeling, IL, United States) to secure the tripolar electrode to the cranium. An Ag/AgCl grounding wire (4 cm long, diameter of 1 mm) was inserted below the skin, with the tip located at the base of the neck. Prior to the transfer to the MRI system, anesthesia was switched to urethane (four consecutive intraperitoneal injections with the dose of 1.25–1.50 g/kg of body weight, 15 min apart) while gradually decreasing the isoflurane level and discontinuing it at the last urethane injection. Optical rectal temperature probe and pressure respiration sensor were employed to monitor body temperature and respiration (Small Animal Instruments Inc., New York, NY, United States), respectively, during the MRI scan. The body temperature was maintained at the level of 37°C using heated water circulation and heated air.

### MRI Acquisition

All MRI scans were conducted with a 9.4-T 31-cm horizontal-bore magnet equipped with Agilent DirecDRIVE console (Palo Alto, CA, United States) using a quadrature radio frequency coil designed for full rat brain coverage. The coil was composed of ^1^H MRI invisible materials, thus ensuring that no unwanted signal would fold into the Field of View (FOV) from the coil itself. Prior to fMRI, anatomical images were acquired using a Fast Spin-Echo (FSE) sequence: repetition time (TR) = 3 s, effective echo time = 48 ms, number of echoes = 8, matrix size = 192^2^, FOV = 3.2 × 3.2 cm^2^, slice thickness = 1 mm, number of slices = 15, no interslice gap and number of averages = 4. Next, MB-SWIFT fMRI was conducted using the following parameters: TR = 0.97 ms, 3094 spokes per volume, resulting in temporal resolution of 3 s, bandwidth (BW) = 192 kHz, matrix size = 64^3^, FOV = 3.5 × 3.5 × 6.4 cm^3^ and flip angle = 6°. Excitation was performed with a chirp pulse gapped into four 2.6-μs sub-pulses (Idiyatullin et al., 2006, 2015). Two-fold oversampling was used during acquisition in the gaps of 32/BW duration. The post-correlation FID (Idiyatullin et al., 2006) consisted of 32 points. MB-SWIFT was chosen instead of conventional Echo Planar Imaging (EPI) as it provides images virtually free of magnetic susceptibility artifacts (Figure 1C) at high magnetic field of 9.4 T caused by the implanted electrode and it does not require magnetic field distortion correction (Lehto et al., 2017a).

### Functional Paradigms of Deep Brain Stimulation

All stimulation paradigms consisted of three blocks of 60 s of rest and 18 s of stimulation, ending with an additional rest period, resulting in 4 min 54 s of total paradigm. To avoid adaptation to stimulus, 2-min breaks were taken between trials. To seek for stimulation frequency (*n* = 6) with the strongest fMRI response, monopolar, biphasic symmetric 180-μs square pulses without interphase delay were delivered, with total current of 1.4–1.7 mA distributed equally among the three electrode channels. The current was chosen as the minimal current that produced amygdala activation. The tested stimulation frequencies were 20, 35, 70, 100, 130, 160, and 200 Hz in randomized order.

OSS was achieved by controlling the orientation of an electric dipole under the tip of the electrode. As the strongest electric field gradient of a dipole is aligned with its primary axis, an axon is the most excitable when the primary axis of a dipole is aligned with the axon (Rattay, 1989; Lehto et al., 2017b). The orientation of the dipole was controlled by changing the current amplitudes of the individual channels relative to each other by choosing the amplitudes from phase offset sinusoids (Lehto et al., 2017b). The OSS was applied using the same square pulses and current amplitudes as described above with a stimulation frequency of 20 Hz (*n* = 8) based on the results of monopolar stimulation with different stimulation frequencies. The stimulation angles were chosen in 30° steps resulting in 12 separate OSS experiments. The angles of stimulation were set such that 0°/180° corresponded to the mediolateral direction and −90°/90° corresponded to rostrocaudal direction (Figure 1B).

The stimulation waveforms were computed and delivered using MATLAB 2016a (Mathworks; Natick, MA, United States) through National Instruments digital-to-analog converter (cDAQ-9174 chassis, 9263 analog output module, 9402 digital input/output module; Austin, TX, United States) and three stimulus-isolators (A-M Systems; Carlsborg, WA, United States) ran as current sources enabling the same peak current through each contact regardless of potential inter-contact impedance differences. The analog output module was used to drive the stimulus isolators (voltage to current conversion), while the digital input/output module was used to software trigger a MATLAB based stimulation script by detecting a TTL voltage from the scanner hardware with the onset of MB-SWIFT MRI pulse sequence (Lehto et al., 2017a). Analog high-pass filters with cut-off frequencies of 0.4 Hz (A-M Systems; Carlsborg, WA, United States) were attached to the outputs of the stimulus-isolators to remove DC drift in the stimulus-isolator output voltages. The stimulus isolators were connected to the electrodes via three 10 m coaxial cables (RG223/U; Pasternack Enterprises, Irvine, CA, United States) and routed through a radiofrequency low-pass filter plate into the MRI scanner Faraday cage to reduce radiofrequency noise of the MRI acquisition. The ends of the three coaxial cables were stripped exposing the shielding and the center conductor. The shielding was soldered to a small copper plate connected to the aforementioned subcutaneous Ag/AgCl ground electrode. The center conductors were soldered to the electrode connector (PlasticsOne, 335-000; Roanoke, VA, United States). Diagram of the stimulation system is shown in Figure 1D.

### MRI Data Processing and Analysis

**FIGURE 2:**
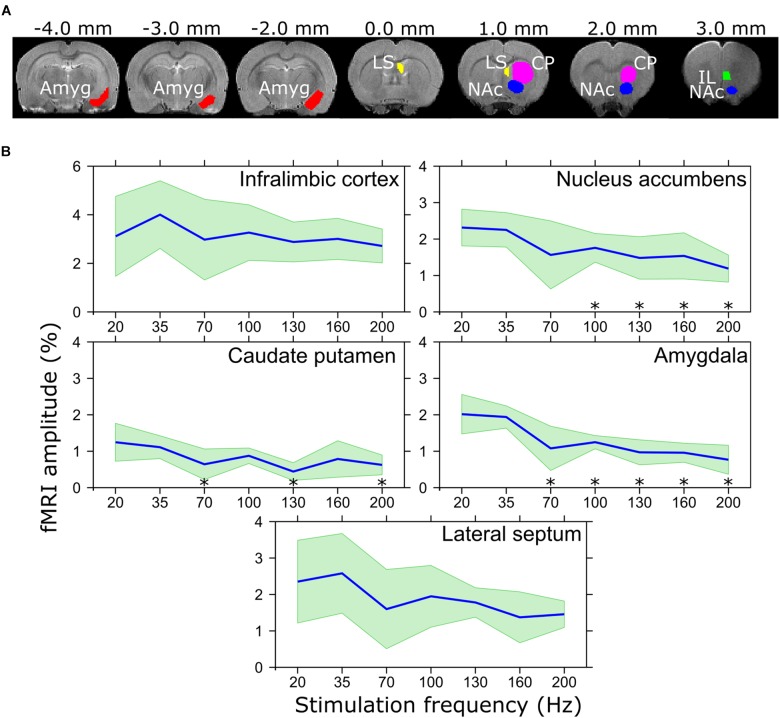
**(A)** Regions of interest representing the IL, NAc, CP, amygdala, and LS, and **(B)** corresponding BOLD amplitudes in response to different stimulation frequencies. ^∗^*p*_FDR_ < 0.05 mean amplitude is smaller than that of 20 Hz stimulation frequency, paired *t*-test. Mean values are shown using blue lines and the standard deviation is shown with green filling.

MB-SWIFT images were reconstructed using gridding and iterative fast iterative shrinkage/thresholding algorithm (Beck and Teboulle, 2009) with three iterations. The resulting data were analyzed in SPM8^1^ and MATLAB 2013b. The pre-processing included motion correction, smoothing with a [2 2 1] pixel FWHM Gaussian kernel, and coregistration and normalization to an animal without an electrode outside the fMRI group based on FSE images. The general linear model consisted of a block design model convolved with a rat hemodynamic function (Silva et al., 2007) and the baseline. For individual analysis, threshold for statistical significance of the activation maps were set to *p* < 10^−5^ (family-wise error corrected). Due to the significant interindividual differences and relatively limited size of the cohort, the standard second level analysis as implemented in SPM8 was not performed. Instead, for group analysis the overlap maps were created (Spiridon et al., 2006), where for each voxel, after coregistration and normalization, the fraction of animals with a statistically significant activation at individual level was calculated. Aggregates of MB-SWIFT fMRI data were then obtained in anatomically defined regions of interest (ROIs) drawn manually with Aedes^2^ based on a rat brain atlas (Paxinos and Watson, 1998). The ROIs were chosen based on the major clusters found during monopolar IL stimulation in the functionally and anatomically relevant areas according to the main hypothesis and included the IL, NAc, CP, and amygdala (Figure 2A). For each ROI and condition, the time series of all voxels in an individual ROI were averaged and the corresponding fMRI response amplitude was assessed by averaging the peak values of the three stimulation periods from the ROI mean time series. On the other hand, the extent of activation was estimated by the number of activated voxels inside the ROIs as calculated from the individual *t*-maps using the individual thresholds for the *t*-values.

Statistical analysis was conducted using one tailed paired *t*-test to compare fMRI amplitudes of monopolar 20 Hz stimulation to those acquired with other frequencies and to compare the OSS angle with the highest mean response to the other stimulation angles. Wilcoxon signed-rank test was used correspondingly to compare the mean number of activated voxels. For one animal OSS angle 90° was missing, and for one animal a single missing frequency dataset (70 Hz) was imputed by the mean of the dataset of other five animals. Correction for multiple comparisons was conducted using false discovery rate (FDR) at ROI level.

After fMRI, rats were sacrificed using a pentobarbital overdose (100 mg/kg; Fatal-Plus, Dearborn, MI, United States). The head was harvested by decapitation and immersion fixed and stored in 10% formalin. The brains were extracted, post-fixed in 10% formalin followed by 10% sucrose. Brains were mounted on to a cryostat for cutting coronal sections at a thickness of 10 μm. Brain tissue sections were placed on to glass slides, and stained with hematoxylin and eosin for visualization of the electrode tract. Finally, confirmation of the implantation of the electrodes into the IL was assessed by light microscopy independently by three experienced researchers.

## Results

The electrode tips were verified to be situated in the IL. Responses to the IL stimulation in individual rats are illustrated in Supplementary Figure 1. In general, the stimulation activated widespread networks or brain regions known to be connected with the IL. These included the local mFC (PL, IL), medial orbitofrontal cortex, anterior cingulate cortex, anterior insula, olfactory tubercle and piriform cortex, basal forebrain structures (bed nucleus of stria terminalis, substantial innominata, diagonal band of Broca), LS, amygdala (basal and cortical nuclei), ventral hippocampus and entorhinal cortex. In addition, we found a robust activation in NAc, which receives strong projections from PL but not IL. From these, the brain regions with the most robust responses were chosen for the ROI analysis including the IL itself, NAc, CP, LS, and amygdala. The ROI analysis of the fMRI response using different stimulation frequencies (Figure 2B) revealed that while the extent of activation did not statistically differ among different frequencies, maximum amplitude of the activation in amygdala was achieved in the lower frequency range (20 and 35 Hz) with a clear declining pattern in higher frequencies and statistically significantly smaller amplitude using 70 (*p*_FDR_ = 0.014), 100 (*p*_FDR_ < 0.001), 130 (*p*_FDR_ < 0.001), 160 (*p*_FDR_ < 0.001), and 200 Hz (*p*_FDR_ < 0.001) as compared to 20 Hz. Similar results were observed in NAc where 100 (*p*_FDR_ = 0.048), 130 (*p*_FDR_ = 0.048), 160 (*p*_FDR_ = 0.048), and 200 Hz (*p*_FDR_ = 0.029) resulted in smaller responses as compared to 20 Hz, while the amplitude in CP was only lower using 70 (*p*_FDR_ = 0.037), 130 (*p*_FDR_ = 0.006), and 200 Hz (*p*_FDR_ = 0.029). In the IL and LS, a similar trend of lower response with higher stimulation frequencies was detected, while no statistically significant differences were found.

**FIGURE 3:**
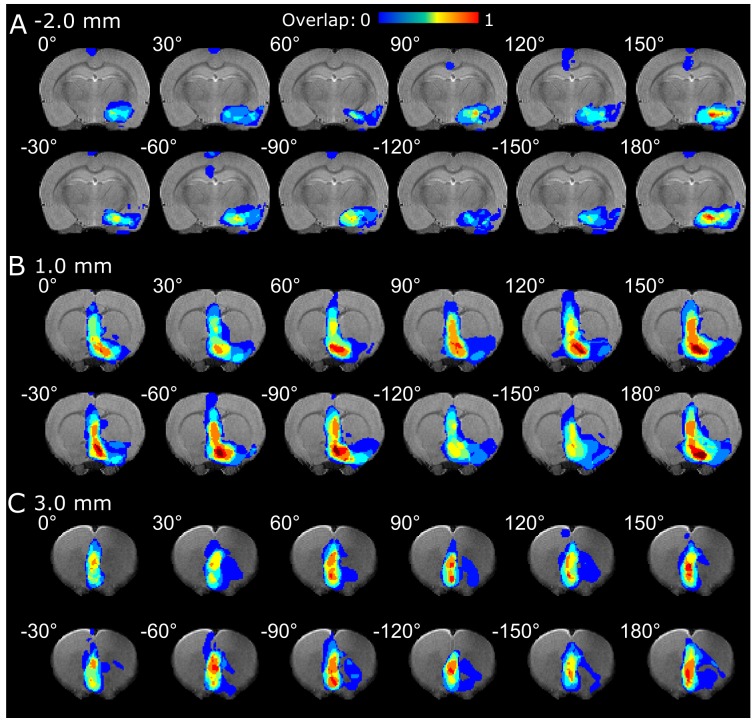
fMRI overlap maps summarizing the responses of the **(A)** amygdala, **(B)** CP, LS and NAc, and **(C)** mFC to OSS of the IL upon different orientations of the OSS.

In the analysis of OSS-fMRI overlap maps, a clear dependence on the angle of stimulation was observed. Multiple angles led to an activation in the amygdala (Figure 3A) and CP, NAc, and LS (Figure 3B), with varying extent of activation and a maximum in the range of 150° and 180°. On the other hand, the activation in the mFC in the vicinity of the electrode exhibited no significant OSS dependence (Figure 3C). On the individual level, one animal out of the eight exhibited negative responses in the thalamus using 60°, 90°, −120°, and −30° stimulation angles (slice not shown).

**FIGURE 4:**
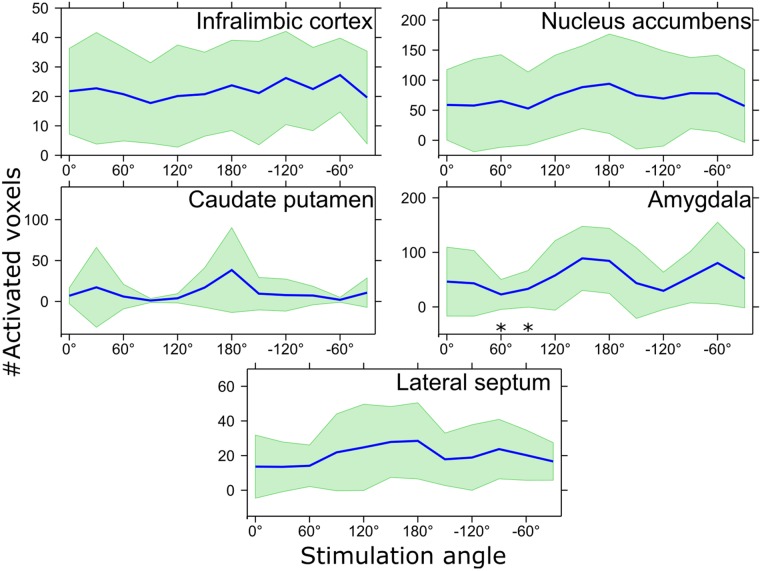
The number of activated voxels in response to various stimulation angles inside the ROIs shown in Figure 3. ^∗^*p*_FDR_ < 0.05 mean number of activated voxels is smaller than that of 150° stimulation angle, Wilcoxon signed-rank test. Mean values are shown using blue lines and the standard deviation is shown with green filling.

The ROI analysis of the OSS-fMRI responses confirmed statistically significant differences in the number of activated pixels observed with different orientations, although differences in amplitude of the fMRI responses did not reach statistical significance. In particular, the number of activated voxels in different ROIs (Figure 4) showed clear orientation dependence in the amygdala, with the main peak at 150° having statistically significantly higher value compared to angles 60° and 90° (*p*_FDR_ = 0.043), while trending at 30°, 120°, –150°, and –120° (*p*_FDR_ = 0.051). Maximum response at 150° showed 89 ± 59 activated voxels whereas minimum at 60° had 23 ± 27 activated voxels. Standard deviation of the number of activated voxels was also clearly higher at the angle of −60°, indicating inter-animal differences in response to OSS. Although OSS led to a clear trend of orientation dependence of the activation, no statistically significant differences were reached in CP (0°, 60°–120°, −90°, −60°; *p*_FDR_ = 0.086) or LS (0°–60°, −120°, −60°, −30°; *p*_FDR_ = 0.086), while the IL and NAc showed virtually no dependence on stimulation angle with no statistically significant differences.

## Discussion

The main rationale for the present study was not only to delineate the neural network activated during IL DBS in rats, but also, and more importantly, to investigate an advanced neuromodulation strategy that can exceed standard DBS programming approaches employed in clinical practice. The complexity of virtually all conceivable MDD DBS targets necessitates highly discriminatory modulation of desired, clinically relevant pathways, while ideally evading various high-risk neural circuits.

Rodent IL, a viable correlate of human sgACC (Diorio et al., 1993; Ostrander et al., 2003), has been reported to receive afferent projections from other medial frontal structures, insula, claustrum, basal forebrain (substantia innominata, diagonal band of Broca, LS), midline thalamic nuclei, hypothalamus, basal amygdala, ventral hippocampus, perirhinal and entorhinal cortex and some midline brainstems structures, including the serotonergic raphe nuclei (Hoover and Vertes, 2007). IL sends efferent projections to medial and orbital frontal cortex, insula, olfactory forebrain, basal forebrain (especially to bed nucleus of stria terminalis), medial thalamus, hypothalamus, basal and medial nuclei of amygdala, and parabrachial and solitary nuclei of brainstem (Saper, 1982; Hurley et al., 1991; Vertes, 2004, 2006). This is consistent with the observed pattern of fMRI activation in the present study with two notable exceptions. First, we found a strong activation in NAc which should not receive major projections from IL. This may result from simultaneous activation of nearby PL with strong projections to NAc (Vertes, 2004) or potentially from current spread. Second, we found no activation in mediodorsal thalamic nucleus which had strong bidirectional connections with IL. Furthermore, the anatomical projections and cytoarchitectural characteristics of IL exhibit significant homologies to clinically relevant human correlates and its stimulation elicits antidepressant-like effects in various behavioral testing paradigms (Hamani et al., 2010b, 2014; Rea et al., 2014), providing both predictive and construct validity of this animal model for translational DBS studies.

Deep brain stimulation effects have been initially explained as a mere functional inactivation of stimulated targets (Benabid et al., 1998; Okun and Vitek, 2004). However, according to the most recent findings, DBS invokes neuromodulation also in brain regions distant from the stimulated target due to the release of various neurotransmitters (Nambu and Chiken, 2015; Florence et al., 2016). These distant effects, specifically selective antidromic stimulation of afferent axons, have even been implicated as the major drivers behind the clinical efficacy of subthalamic nucleus DBS in movement disorders (Gradinaru et al., 2009). Analogous results have been seen as markedly good antidepressant-like effect of IL DBS even after destroying neuronal bodies and sparing axons using ibotenic acid in a rat model (Hamani et al., 2010b). This major role of axons in the DBS effect in IL is of utmost importance for our advanced OSS paradigms capable of angle-dependent axonal stimulation based on the direction of electrical field gradient (Lehto et al., 2017b), thus providing a further dimension for the optimisation and individualisation of DBS parameters.

The exact mechanisms underlying antidepressant effects of various possible DBS targets are still obscure. Partially overlapping but also different neural circuits are likely modulated when stimulation is applied to different common DBS target areas (Hamani et al., 2014). Indeed, IL DBS in rats was shown to reduce anhedonia-like behavior (Rea et al., 2014) and exert anxiolytic effects (Edemann-Callesen et al., 2015), while e.g., DBS of medial forebrain bundle significantly interacts also with the reward system (Edemann-Callesen et al., 2015). Targeting specific DBS parameters, including the stimulation frequency of the individual nodes, advanced electrode configurations (Martens et al., 2011; Tsai et al., 2015) and OSS (Lehto et al., 2017b), might be highly beneficial for eliciting desired outcomes. Even though the therapeutic relevance of the circuits recruited in the present study could not be directly tested due to the use of normal, healthy rats, and the translational nature of animal model networks to psychiatric disorders is also indirect, preclinical models allow us to appraise the activation patterns specific to advanced DBS paradigms in comparable settings.

While the fMRI results of this initial study on rats are highly promising in demonstrating that novel DBS paradigms can effectively modulate distinct circuitries without changing the stimulation sites, any inference on clinical effects would be an oversimplification, neglecting differences between two distinct mammalian species and uncertain correlation of fMRI-detected responses and the desired clinical effect. Moreover, OSS outcomes demonstrated a non-negligible interindividual variability. The origin of this discrepancy may stem from the deviations in the location of the stimulated area, as well as from inherent minor anatomical variability to be expected even in laboratory conditions. Our implantation precision is in the range of previous DBS studies in IL (Hamani et al., 2010a) and outcomes have similar variability as expected in clinical practice. It should be noted that deflections of even mere 0.5 mm in an inherently complex rat mFC with the volume of 1.7–1.9 mm^3^ (Hamani and Nobrega, 2012) unavoidably lead to different responses in individual animals, which could eventually be attenuated using a more advanced, high-density electrode design corresponding to the spatial constraints of the stimulated area and allowing for precise location selection (Tsai et al., 2015). Further caveat must be considered with regard to our results. Bearing in mind the dimensions of the rodent IL and the configuration of our stimulation electrode, including the high currents necessary to overcome fMRI response suppression in anesthetised animals, certain level of current spread seems unavoidable. Notably, comparable current strengths have been successfully implemented in other DBS studies (Dunn et al., 2009; Lai et al., 2014), without any detected electric current related tissue damage. Moreover, the current spill-over to adjacent areas could be of interest as well, since more significant antidepressant-like response was reported in prelimbic (PL) DBS (Hamani et al., 2010a), even though other authors rather associate IL with antidepressant-like functions and relevant projections (Hurley et al., 1991; Gabbott et al., 2003; Edemann-Callesen et al., 2015). Nonetheless, the potential stimulation spread under these current amplitudes prohibits more complex deductions on the specific networks activated during stimulation.

Given the large stimulation spread due to the relatively strong stimulation current and the large size of the electrode in respect to the stimulated area, and high interconnectivity of the regions in the immediate vicinity to the implanted lead, significant OSS effects were not expected in the IL, PL or in the regions with the strongest connections such as NAc, although the trend of sensitivity to OSS was observed in the CP and LS. It was expected that connections to these regions were stimulated at every stimulation angle. On the other hand, the effect of OSS in amygdala was more expected as its connection to the IL is more defined. Previous studies observed the strongest anti-depressant effects in the range of 100–130 Hz (Hamani and Nóbrega, 2010; Lim et al., 2015), however, the aim of using OSS-DBS was to show differential network level responses using fMRI. Hence, monopolar stimulation frequency eliciting the highest fMRI response in the amygdala (20 Hz) was chosen based on preliminary studies. As DBS induces a combination of inhibitory and excitatory effects, strong fMRI response and treatment response do not necessarily go hand in hand. Finally, in our previous work stimulating the corpus callosum (Lehto et al., 2017b), symmetry in the strength of fMRI response was shown between 0° and 180° stimulation angles while in the current study similar effect was not observed. This is likely related to the much more complicated local anatomy of the IL as compared to the corpus callosum and the size of the electrodes used for stimulation. In the corpus callosum, the electrode tip was embedded inside the structure with very well defined fiber orientation in all of its surrounding, while in the IL the orientation distribution and geometry of fibers relative to the electrode is likely much more complex. To better target specific fiber orientations near the electrode, high-density electrode designs are needed with more flexible capability of orienting electric field gradient in space rather than on the plane, which limited in part the effect of the OSS for stimulating IL/PL areas of the brain.

## Conclusion

Our findings show adjustable activation of the rat limbic system when applying OSS to the IL, a homologue to the human sgACC identified as a DBS target for treating MDD. OSS may offer a new avenue for stimulus parameter optimization using DBS so that only relevant pathways are stimulated, while simultaneously avoiding crossing pathways associated with possible adverse events. In combination with pre-implantation MRI could be invaluable for individualization of DBS treatment in complex brain areas such as the sgACC, where OSS strategies could be beneficial for expanding capabilities of DBS. High-density electrodes with clearly smaller contact size and pitch as compared to the present study are likely needed, as they will allow separating small distinct fiber bundles with different orientations. Finally, although our study is a first demonstration of the capabilities of orientation selective DBS in stimulating areas relevant to MDD, further technological developments of multielectrode arrays and additional studies are required to substantiate the potential of our findings as a direction for treatment therapy of MDD.

## Author Contributions

LL and PF participated in design of the work, acquisition, analysis, interpretation of data and preparing the manuscript. HL participated in design of the work, acquisition and preparing the manuscript. AS participated in interpretation of data and preparing the manuscript. JS participated in design of the work and preparing the manuscript. MJ participated in analysis, interpretation of data and preparing the manuscript. LE participated in analysis and preparing the manuscript. WL participated in design of the work, interpretation of data and preparing the manuscript. OG, HT, SMa, and SMi participated in design of the work, analysis, interpretation of data and preparing the manuscript.

## Conflict of Interest Statement

The authors declare that the research was conducted in the absence of any commercial or financial relationships that could be construed as a potential conflict of interest.

## Footnotes

1 http://www.fil.ion.ucl.ac.uk/spm

2 http://aedes.uef.fi

## Abbreviations

Amyg: amygdala
CP: caudate putamen
fMRI: functional magnetic resonance imaging
IL: infralimbic cortex
LS: lateral septum
MB-SWIFT: multi-band sweep imaging with fourier transformation
mFC: medial frontal cortex
NAc: nucleus accumbens
PL: prelimbic cortex
ROI: region of interest
sgACC: subgenual cingulate cortex
