# Supplementary material for: Tuning Neuromodulation Effects by Orientation Selective Deep Brain Stimulation in the Rat Medial Frontal Cortex
Source: Front Neurosci. 2018 Dec 13;12:899. doi: 10.3389/fnins.2018.00899 (PMC6300504; doi:10.3389/fnins.2018.00899)
Supplement: Supplementary file 1 [file Presentation_1.PPTX]

## Slide 1
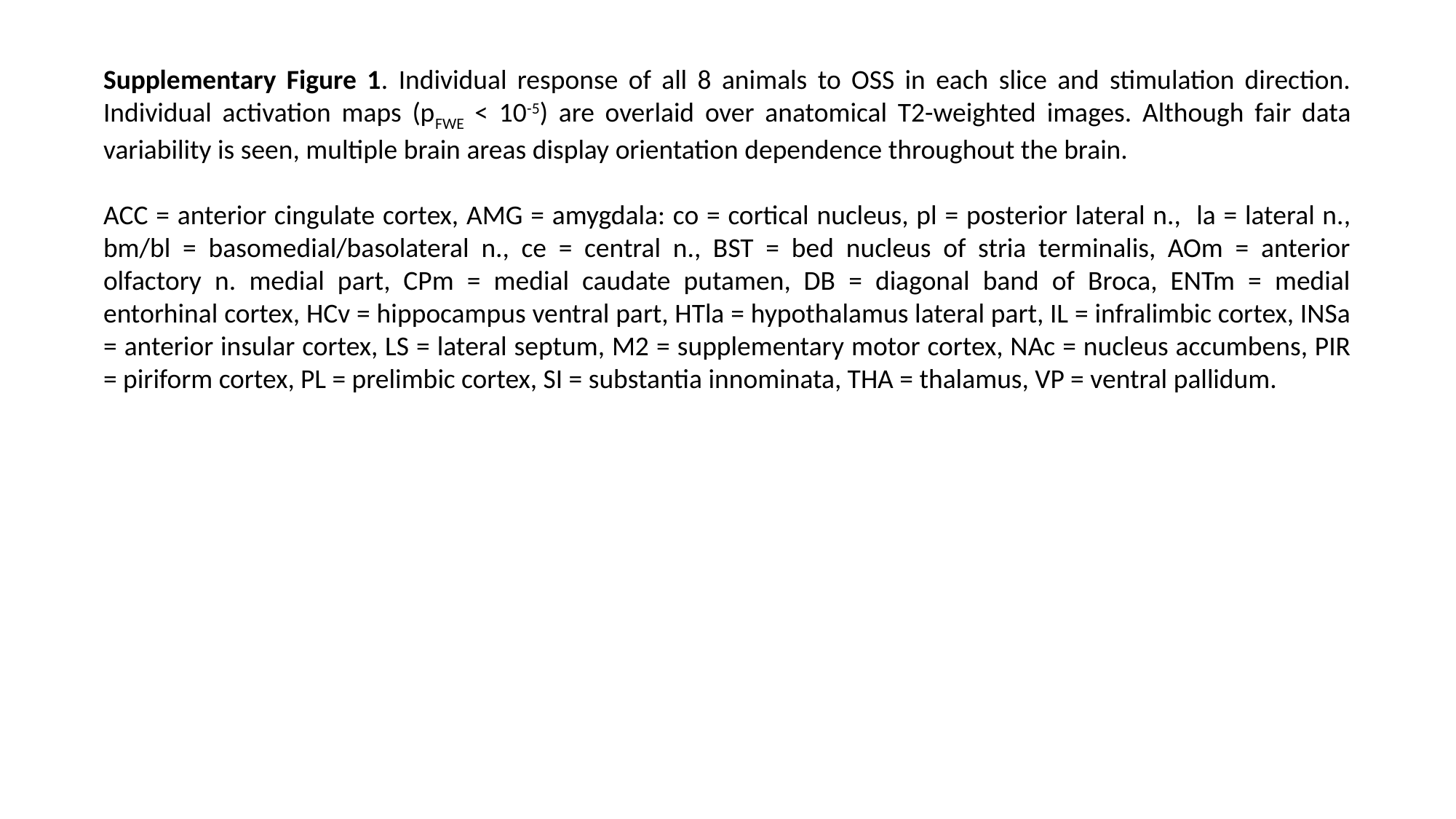

Supplementary Figure 1. Individual response of all 8 animals to OSS in each slice and stimulation direction. Individual activation maps (pFWE < 10-5) are overlaid over anatomical T2-weighted images. Although fair data variability is seen, multiple brain areas display orientation dependence throughout the brain.
ACC = anterior cingulate cortex, AMG = amygdala: co = cortical nucleus, pl = posterior lateral n., la = lateral n., bm/bl = basomedial/basolateral n., ce = central n., BST = bed nucleus of stria terminalis, AOm = anterior olfactory n. medial part, CPm = medial caudate putamen, DB = diagonal band of Broca, ENTm = medial entorhinal cortex, HCv = hippocampus ventral part, HTla = hypothalamus lateral part, IL = infralimbic cortex, INSa = anterior insular cortex, LS = lateral septum, M2 = supplementary motor cortex, NAc = nucleus accumbens, PIR = piriform cortex, PL = prelimbic cortex, SI = substantia innominata, THA = thalamus, VP = ventral pallidum.

## Slide 2
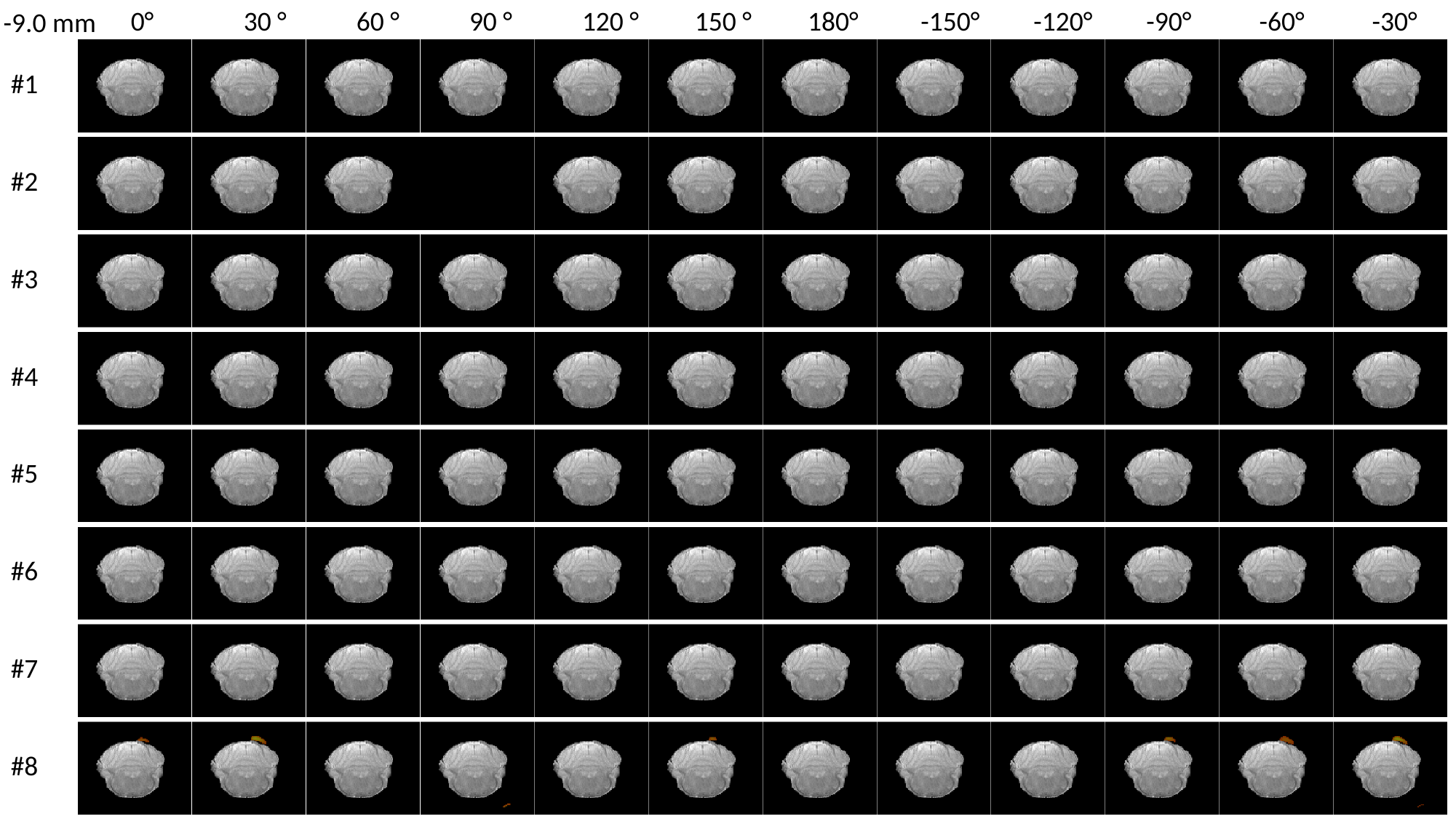

-9.0 mm
0°
30 °
60 °
90 °
120 °
150 °
180°
-150°
-120°
-90°
-60°
-30°
#1
#2
#3
#4
#5
#6
#7
#8

## Slide 3
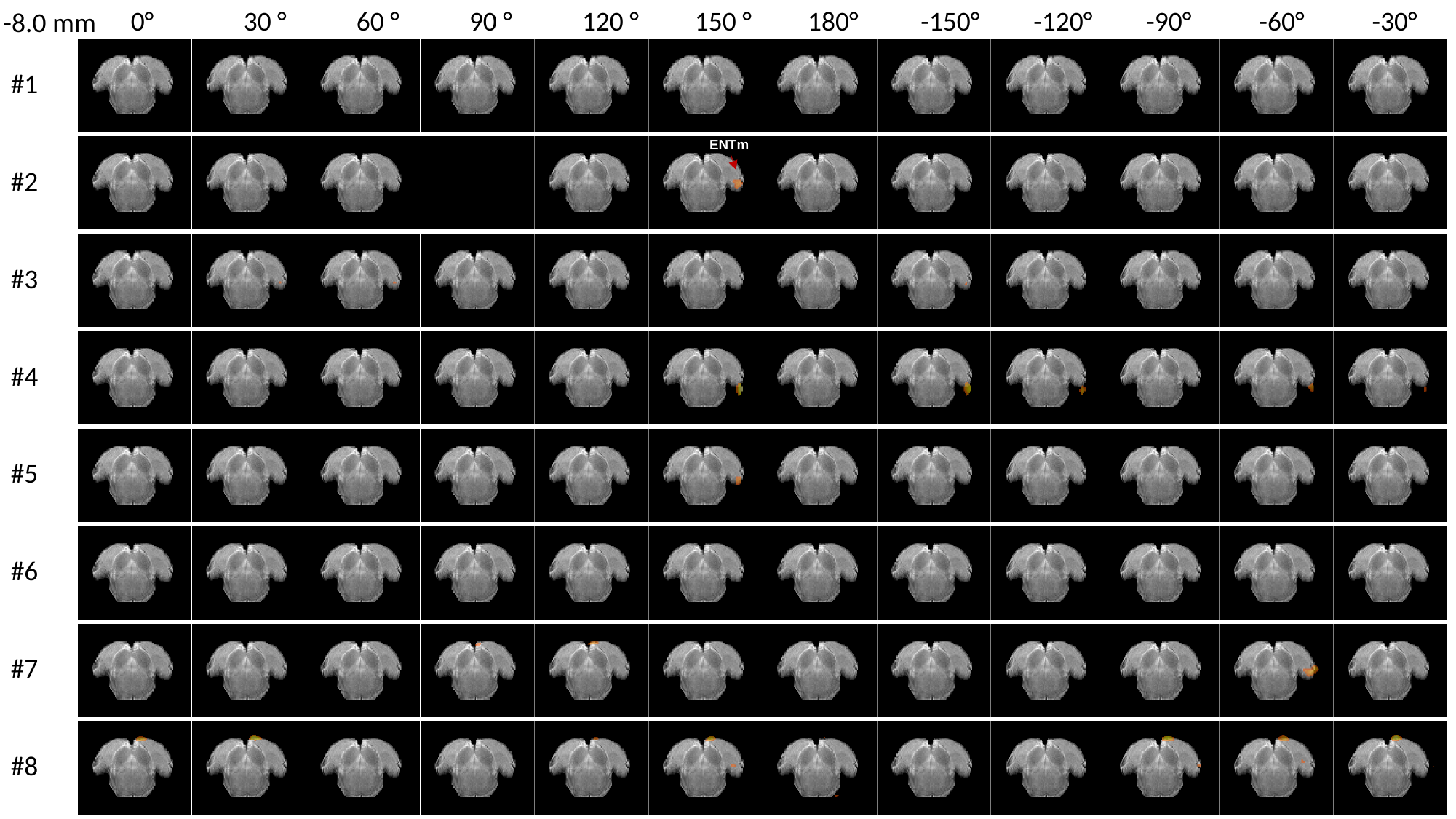

-8.0 mm
0°
30 °
60 °
90 °
120 °
150 °
180°
-150°
-120°
-90°
-60°
-30°
#1
ENTm
#2
#3
#4
#5
#6
#7
#8

## Slide 4
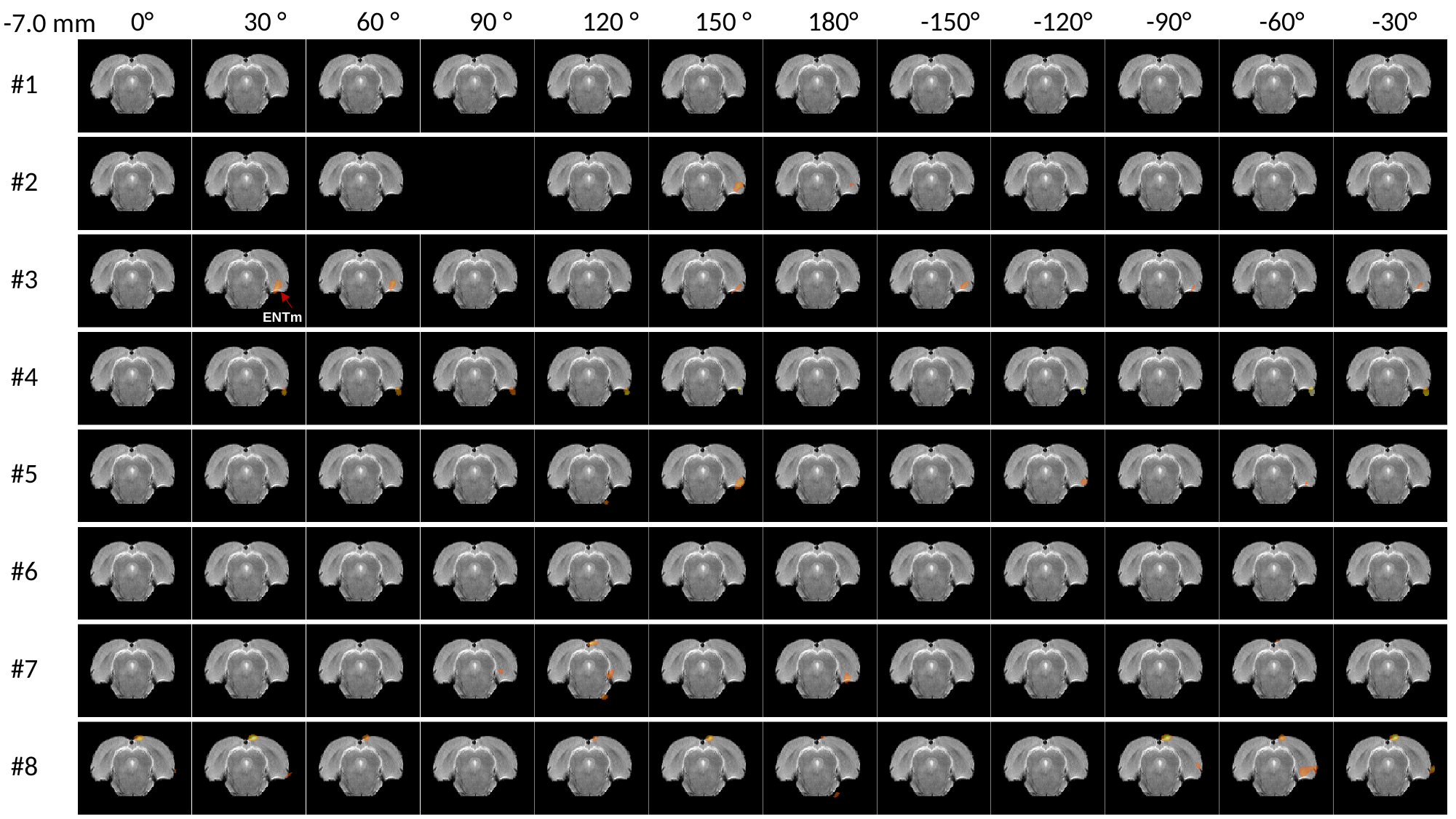

-7.0 mm
0°
30 °
60 °
90 °
120 °
150 °
180°
-150°
-120°
-90°
-60°
-30°
#1
#2
#3
ENTm
#4
#5
#6
#7
#8

## Slide 5
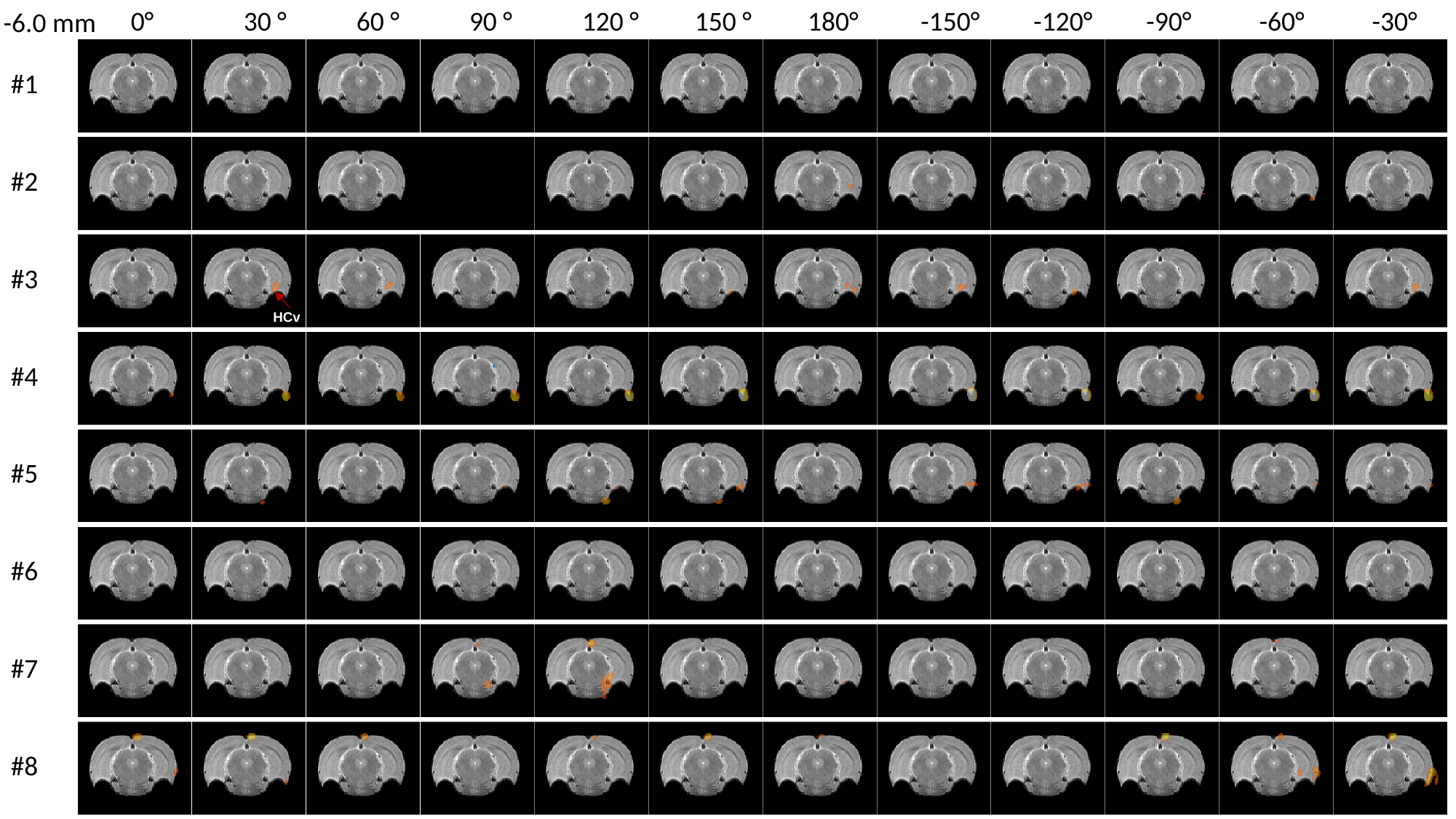

-6.0 mm
0°
30 °
60 °
90 °
120 °
150 °
180°
-150°
-120°
-90°
-60°
-30°
#1
#2
#3
HCv
#4
#5
#6
#7
#8

## Slide 6
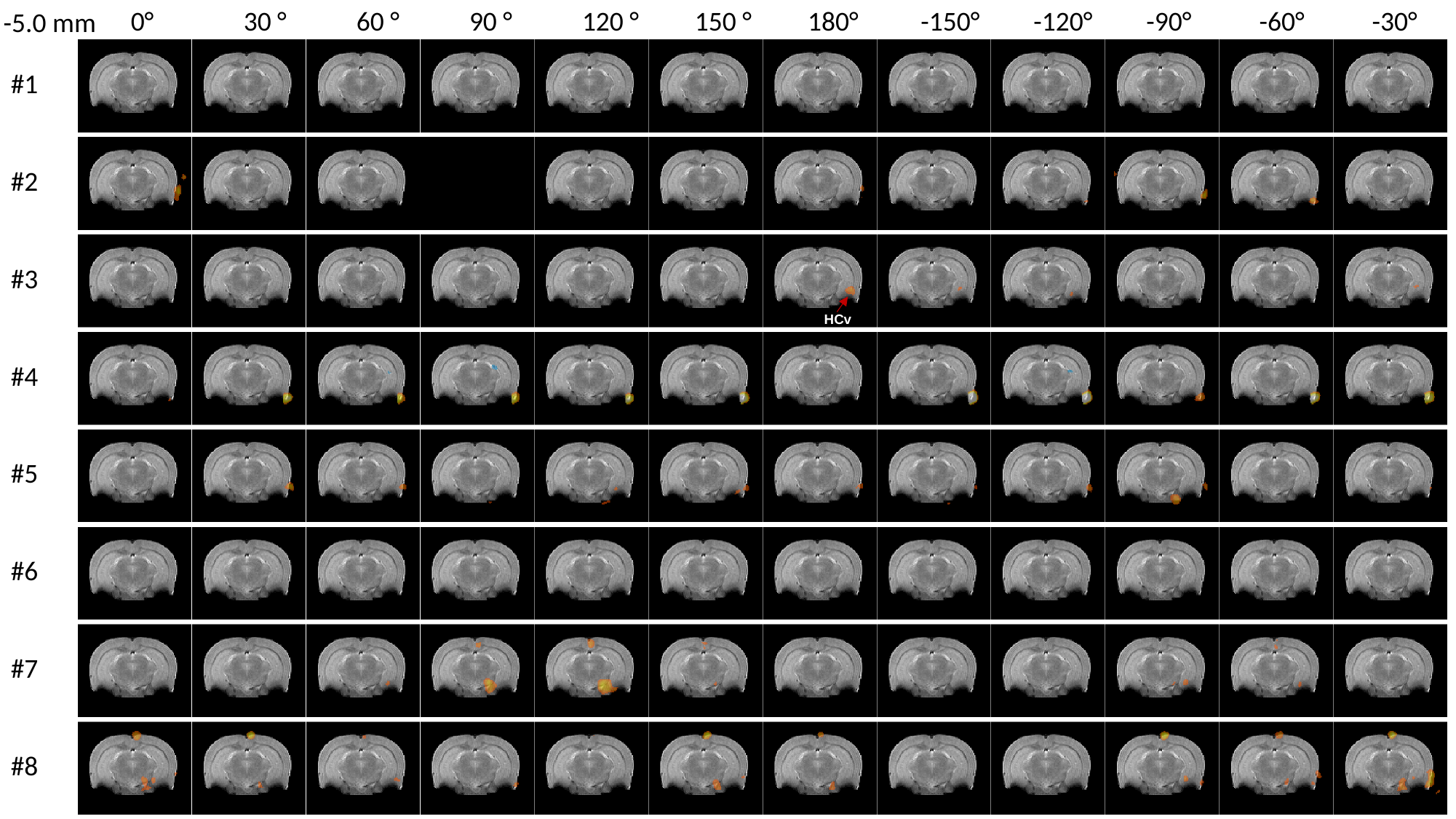

-5.0 mm
0°
30 °
60 °
90 °
120 °
150 °
180°
-150°
-120°
-90°
-60°
-30°
#1
#2
#3
HCv
#4
#5
#6
#7
#8

## Slide 7
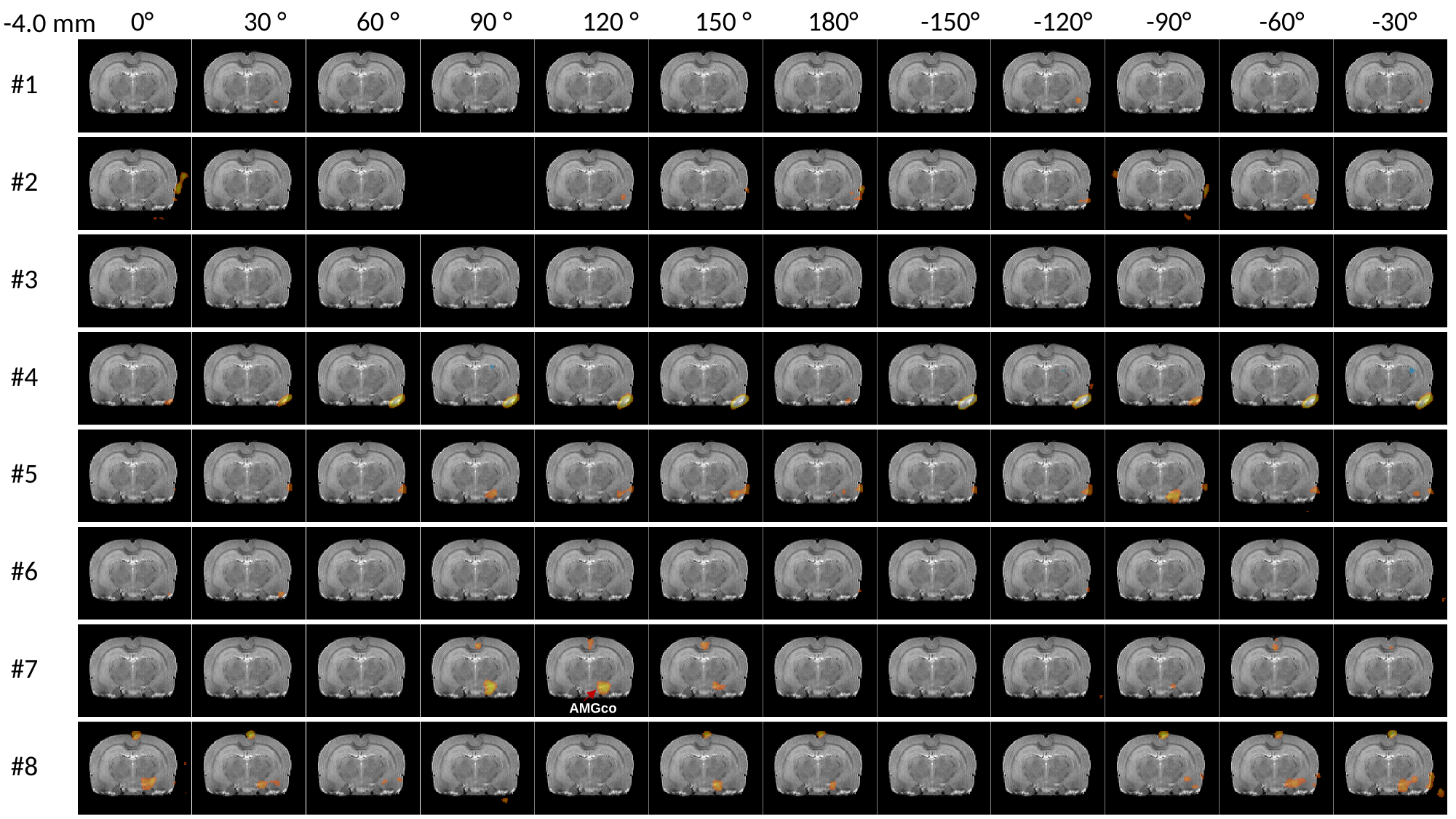

-4.0 mm
0°
30 °
60 °
90 °
120 °
150 °
180°
-150°
-120°
-90°
-60°
-30°
#1
#2
#3
#4
#5
#6
#7
AMGco
#8

## Slide 8
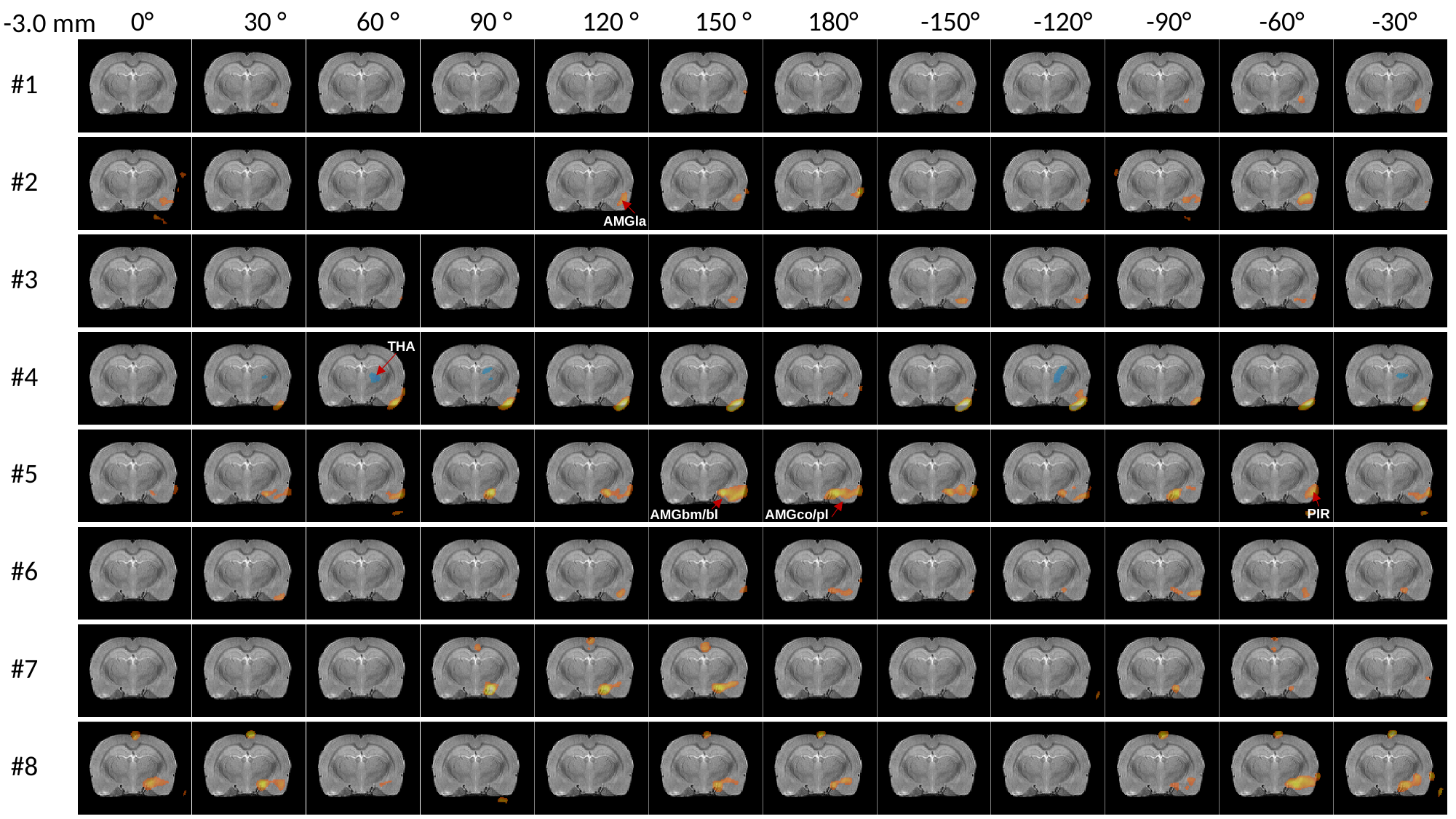

-3.0 mm
0°
30 °
60 °
90 °
120 °
150 °
180°
-150°
-120°
-90°
-60°
-30°
#1
#2
AMGla
#3
THA
#4
#5
PIR
AMGco/pl
AMGbm/bl
#6
#7
#8

## Slide 9
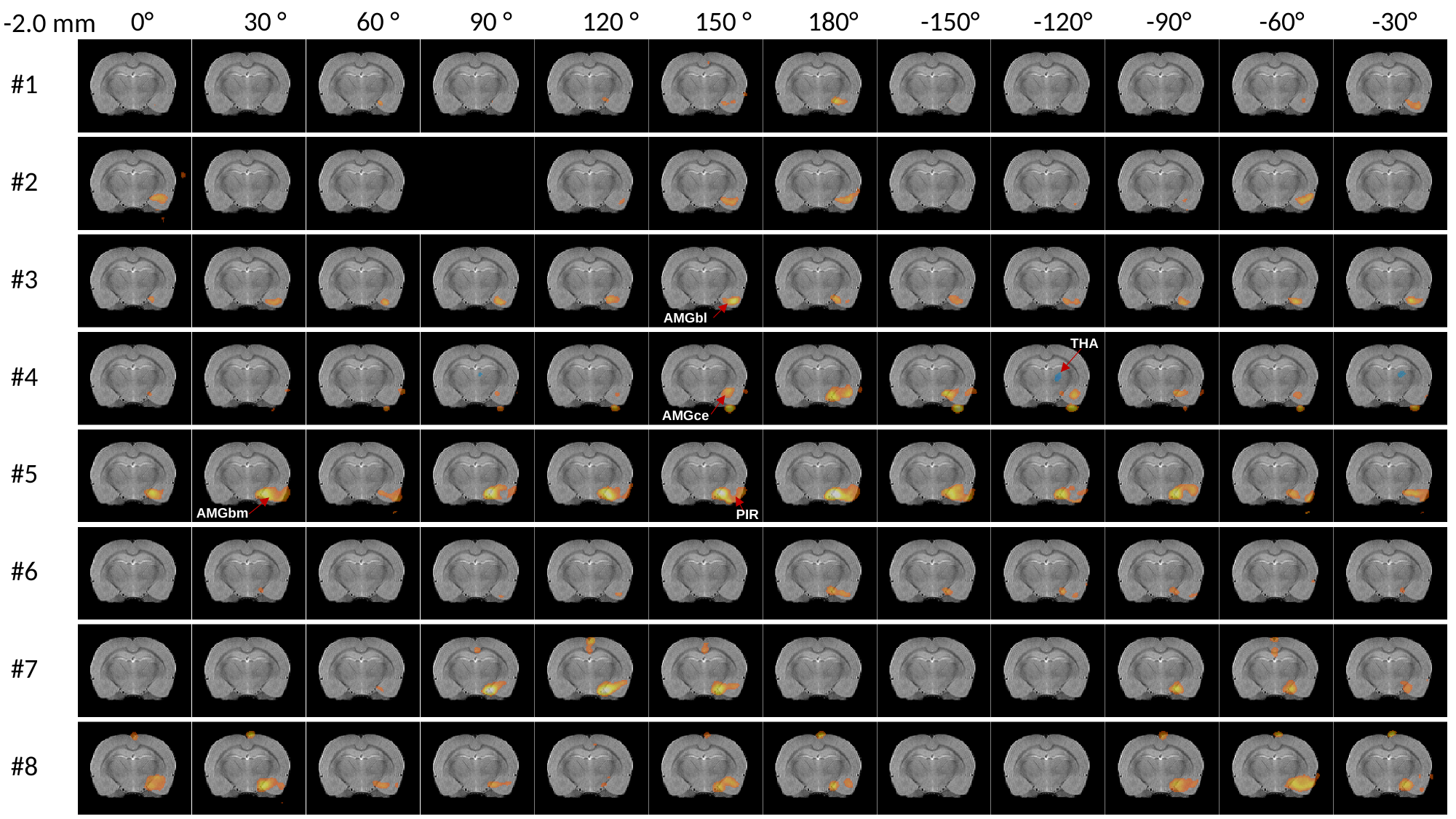

-2.0 mm
0°
30 °
60 °
90 °
120 °
150 °
180°
-150°
-120°
-90°
-60°
-30°
#1
#2
#3
AMGbl
THA
#4
AMGce
#5
AMGbm
PIR
#6
#7
#8

## Slide 10
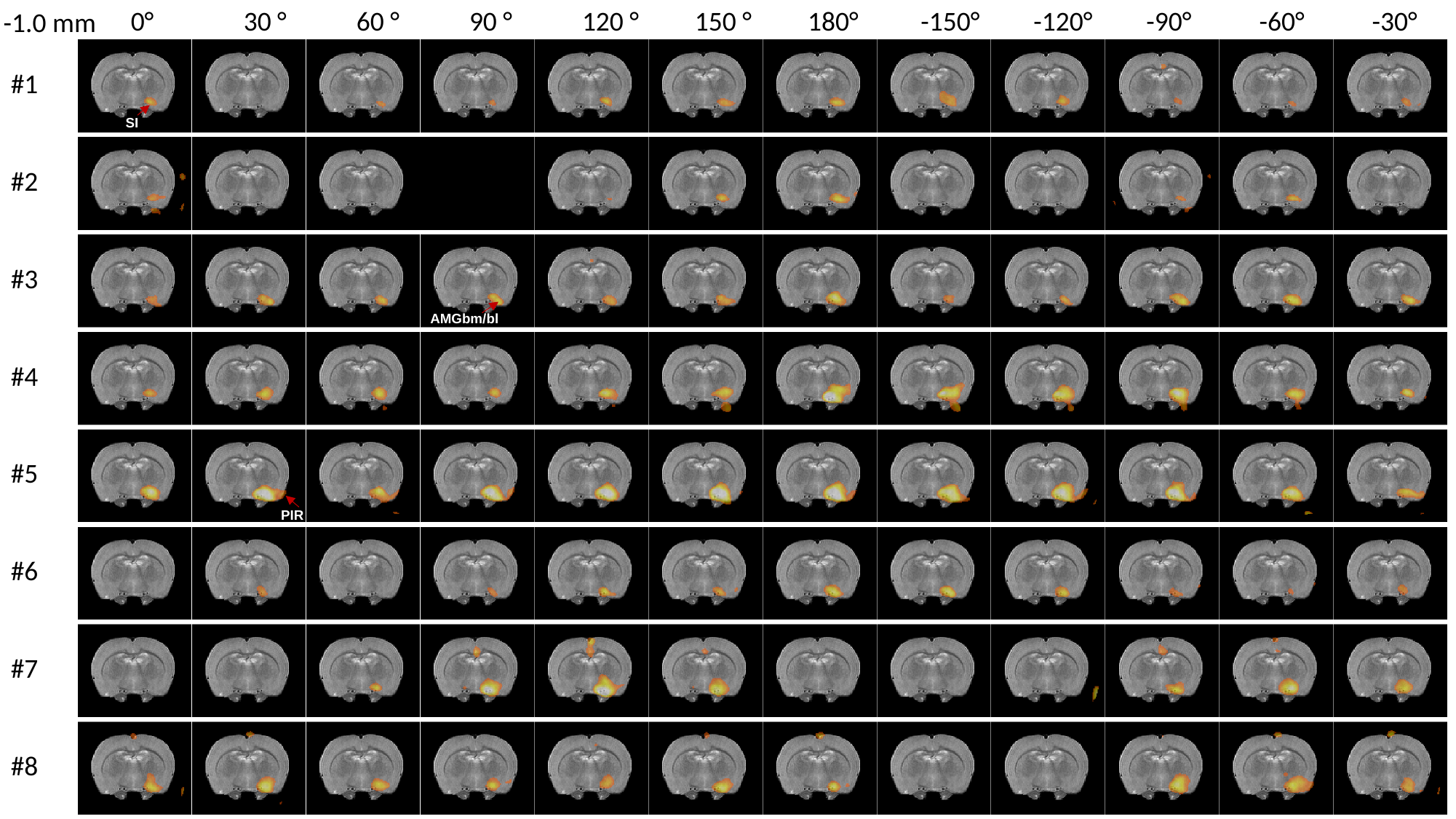

-1.0 mm
0°
30 °
60 °
90 °
120 °
150 °
180°
-150°
-120°
-90°
-60°
-30°
#1
SI
#2
#3
AMGbm/bl
#4
#5
PIR
#6
#7
#8

## Slide 11
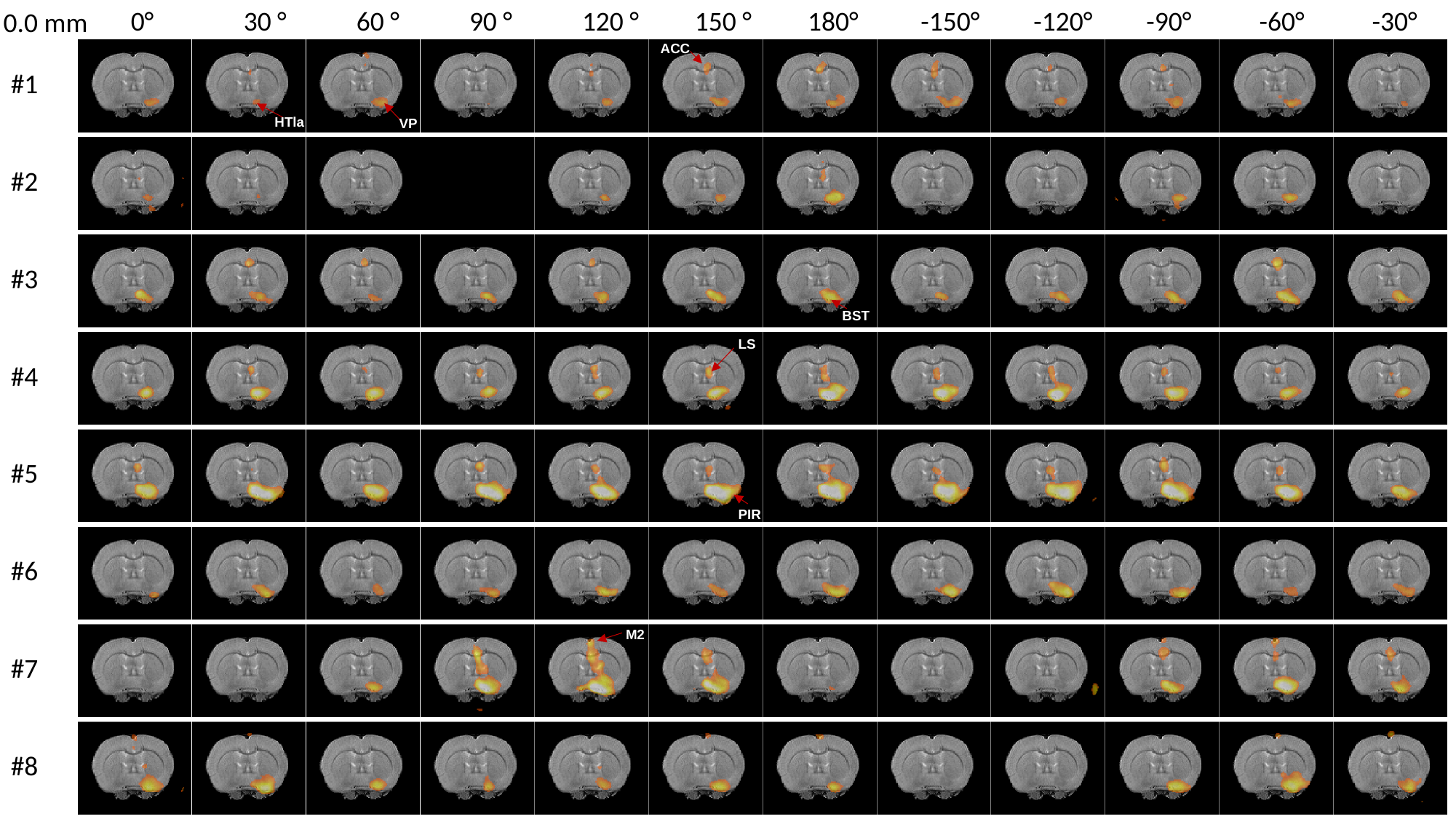

0.0 mm
0°
30 °
60 °
90 °
120 °
150 °
180°
-150°
-120°
-90°
-60°
-30°
ACC
#1
HTla
VP
#2
#3
BST
LS
#4
#5
PIR
#6
M2
#7
#8

## Slide 12
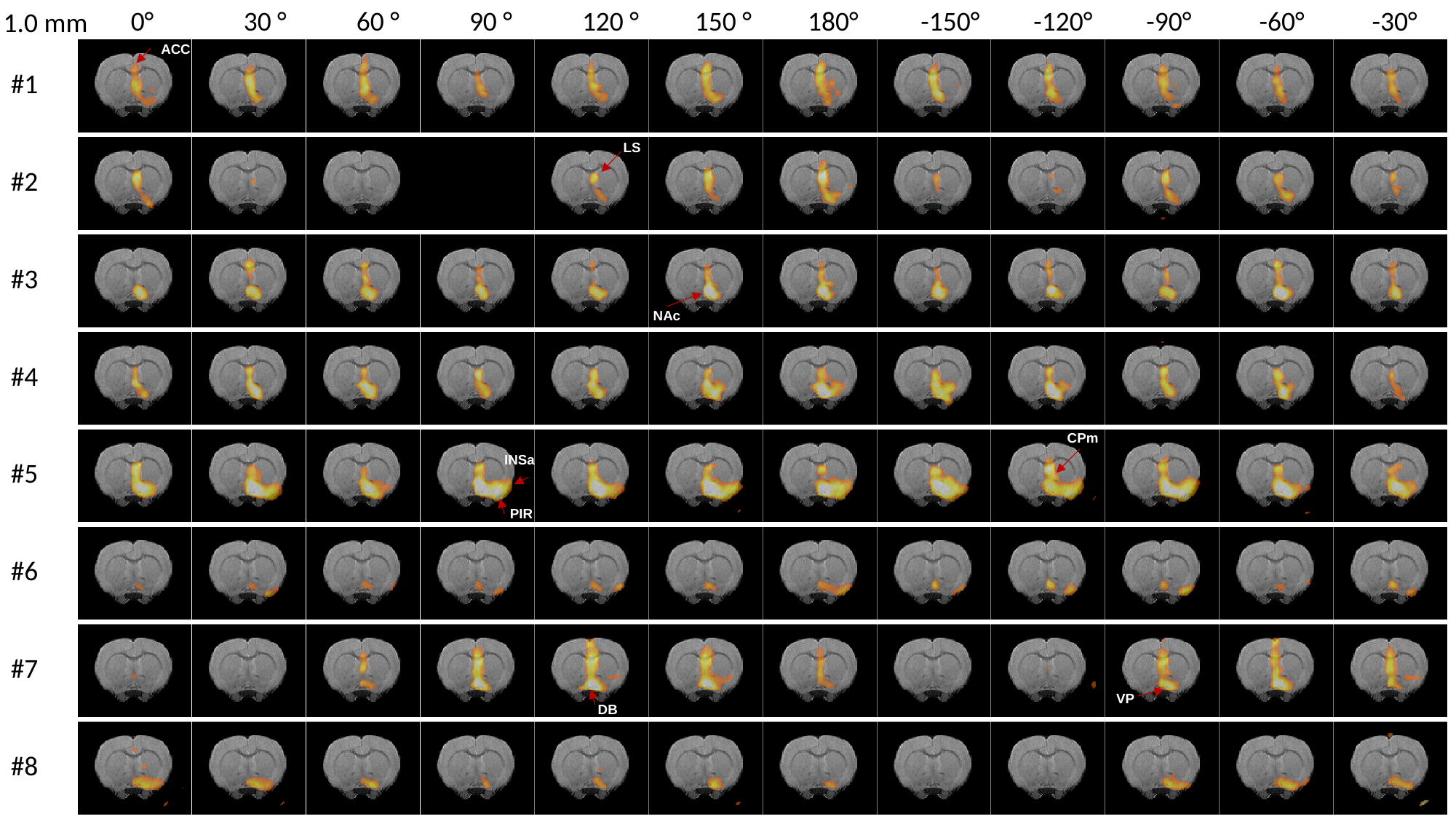

1.0 mm
0°
30 °
60 °
90 °
120 °
150 °
180°
-150°
-120°
-90°
-60°
-30°
ACC
#1
LS
#2
#3
NAc
#4
CPm
INSa
#5
PIR
#6
#7
VP
DB
#8

## Slide 13
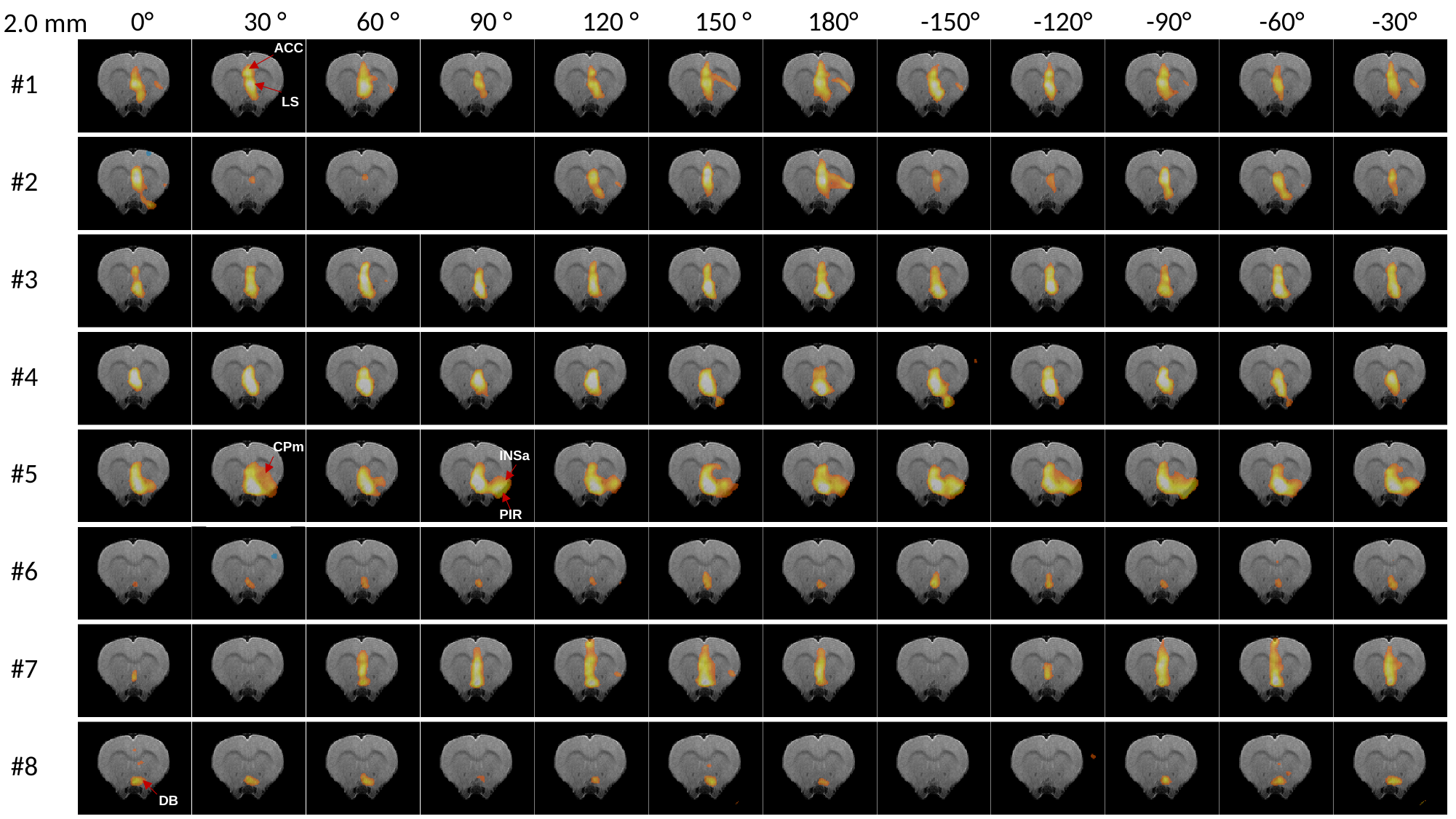

2.0 mm
0°
30 °
60 °
90 °
120 °
150 °
180°
-150°
-120°
-90°
-60°
-30°
ACC
#1
LS
#2
#3
#4
CPm
INSa
#5
PIR
#6
#7
#8
DB

## Slide 14
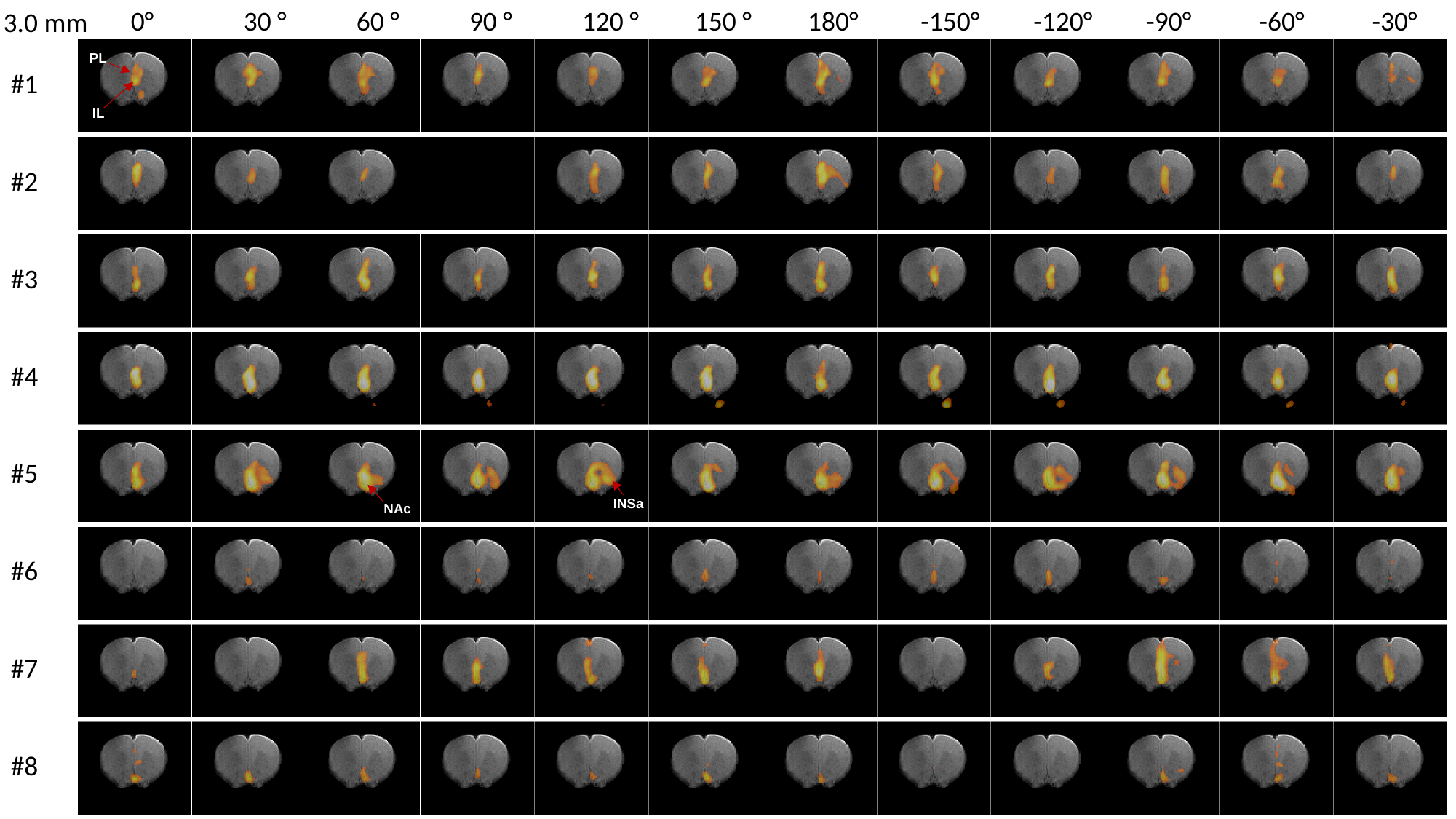

3.0 mm
0°
30 °
60 °
90 °
120 °
150 °
180°
-150°
-120°
-90°
-60°
-30°
PL
#1
IL
#2
#3
#4
#5
INSa
NAc
#6
#7
#8

## Slide 15
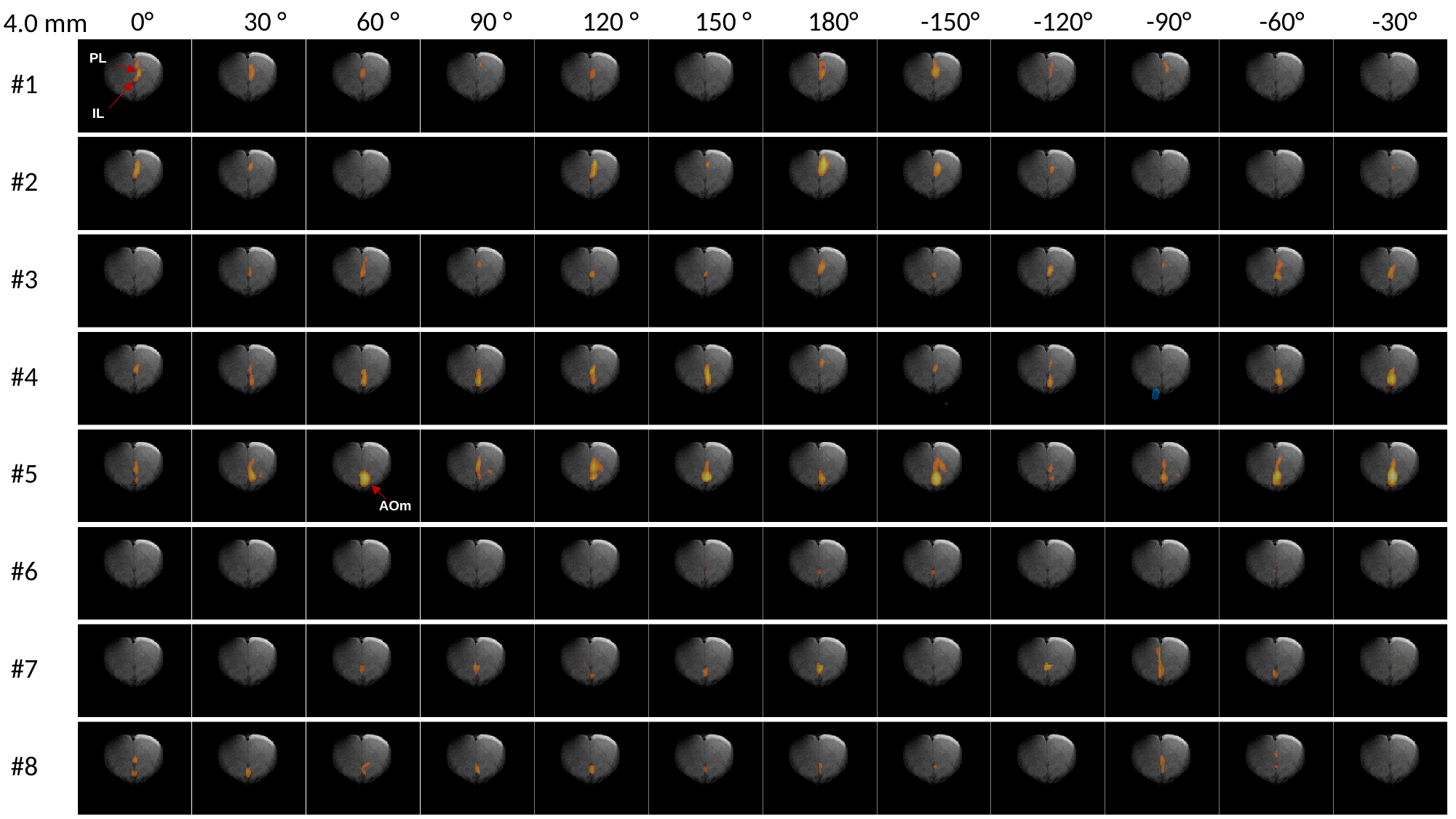

4.0 mm
0°
30 °
60 °
90 °
120 °
150 °
180°
-150°
-120°
-90°
-60°
-30°
PL
#1
IL
#2
#3
#4
#5
AOm
#6
#7
#8

## Slide 16
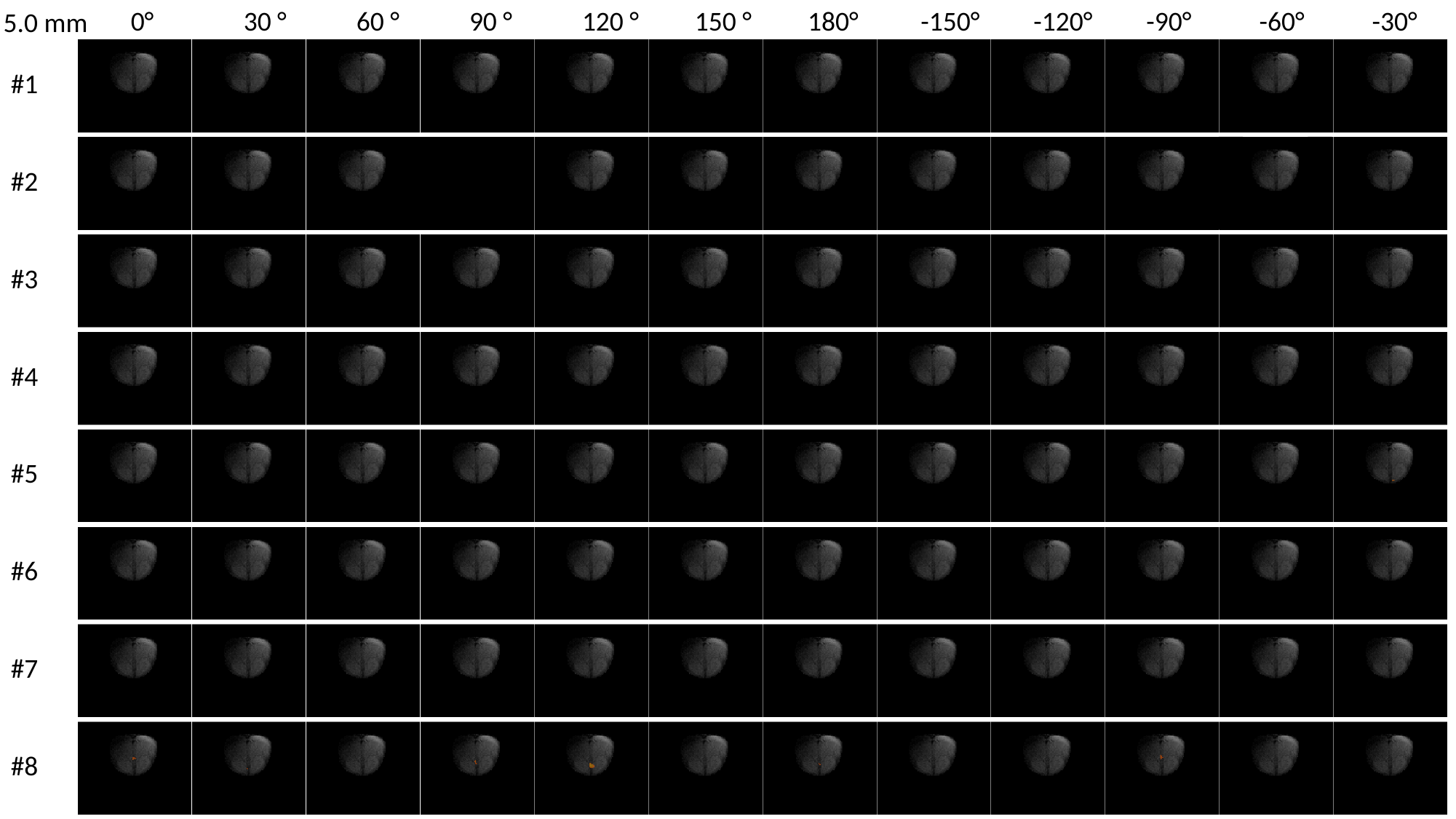

5.0 mm
0°
30 °
60 °
90 °
120 °
150 °
180°
-150°
-120°
-90°
-60°
-30°
#1
#2
#3
#4
#5
#6
#7
#8
